# Solvent-Dependent Characterization of Fucoxanthin through 2D Electronic Spectroscopy Reveals New Details on the Intramolecular Charge-Transfer State Dynamics

**DOI:** 10.1021/acs.jpclett.1c00851

**Published:** 2021-05-17

**Authors:** Giampaolo Marcolin, Elisabetta Collini

**Affiliations:** Department of Chemical Sciences, University of Padova, Via Marzolo 1, I-35131 Padova, Italy

## Abstract

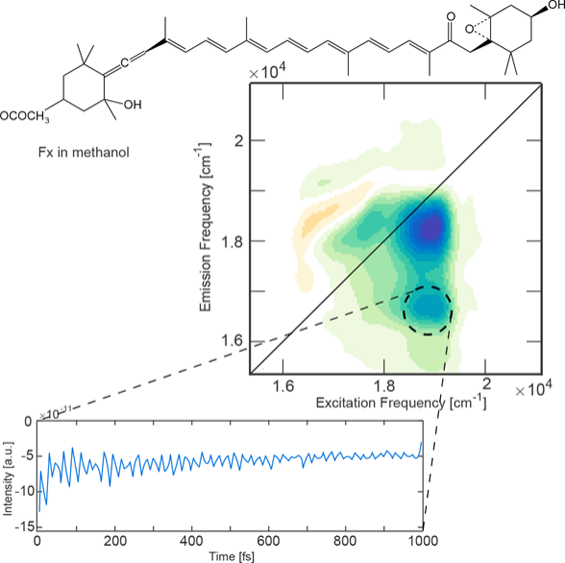

The electronic state manifolds of carotenoids and their relaxation dynamics are the object of intense investigation because most of the subtle details regulating their photophysics are still unknown. In order to contribute to this quest, here, we present a solvent-dependent 2D Electronic Spectroscopy (2DES) characterization of fucoxanthin, a carbonyl carotenoid involved in the light-harvesting process of brown algae. The 2DES technique allows probing its ultrafast relaxation dynamics in the first 1000 fs after photoexcitation with a 10 fs time resolution. The obtained results help shed light on the dynamics of the first electronic state manifold and, in particular, on an intramolecular charge-transfer state (ICT), whose photophysical properties are particularly elusive given its (almost) dark nature.

Carotenoids are pigments that play a crucial role in photoprotection^1−4^ and energy-transfer processes taking place in light-harvesting complexes.^5−11^ It is clear that to understand the fine details of these processes, an accurate description of the photophysics of these molecules is necessary. Many decades of investigations lead to a good knowledge of the main photophysical and dynamic properties of this crucial family of chromophores.

The structural feature common to all the carotenoids is a long polyene backbone of *N* conjugated C=C bonds, a number that determines the main spectroscopic properties of these chromophores.^10,12^ The main challenge in characterizing the electronic states of carotenoids is connected primarily to the presence of dark states, one of their most intriguing features. In fact, it is well-known that the absorption properties of longer polyenes are dominated by the transition from the ground state (S_0_) to the second excited state (S_2_), since the transition to the first excited state (S_1_) is symmetry forbidden.^13−16^ Historically, the dark S_1_ state has been studied, for example, through pump–probe spectroscopy where S_1_ can be populated indirectly from the S_2_ state, through ultrafast internal conversion (typically happening in hundreds of femtoseconds),^17−19^ and then it can be probed thanks to an excited state absorption (ESA) promoted by the transition from S_1_ to higher excited states (S*_n_*). The ESA signal decays as the S_1_ population returns to S_0_, giving information about the S_1_ lifetime (usually characterized by a picosecond time scale).^10,18,20^

In this work, the attention is focused on fucoxanthin (Fx), a carotenoid typically found in diatoms antenna proteins called fucoxanthin-chlorophyll proteins (FCP), that belong to the family of intrinsic light-harvesting complexes. In FCP, fucoxanthin is directly involved in the light-harvesting actions since it serves as a major light-harvesting pigment, transferring excitation energy very efficiently to chlorophyll molecules.^21−24^ It is classified as a carbonyl carotenoid, a class of carotenoids that presents a keto group conjugated with the polyene chain. This functional group is responsible for peculiar spectroscopic features, mainly associated with the formation of an intramolecular charge-transfer (ICT) state in the excited states’ manifold. Carbonyl carotenoids generally display steady-state absorption spectra asymmetrically broadened and devoid of the characteristic vibronic structure. Moreover, their transient absorption spectra are characterized by the presence of an additional ESA band, associated with the ICT → S_*n*′_ transition.^25−32^ These features appear or are enhanced when the system is dissolved in a polar environment that supposedly stabilizes the ICT state. In these conditions, the ICT can be effectively populated via internal conversion from S_2_.^26^ Extensive experimental and computational studies have not succeeded yet to fully clarify the nature and the properties of the ICT state, whether it is coupled with the S_1_ state, in which case the two states would represent two minima of the same potential energy surface,^33−36^ or it is a distinct electronic state.^28,29,31,35,37^ Several models have been suggested, but none of them fully explains all the experimental evidence collected to date.^12,38,39^

A better understanding of the nature and the photophysical behavior of the ICT state is crucial considering the key role it might assume in the excitation energy-transfer mechanisms at the base of the photosynthetic process in the antenna complexes, where the efficiency of the transfer between carotenoids and chlorophylls may exceed 90%.^21,40−42^

In this work, Two-Dimensional Electronic Spectroscopy (2DES) has been exploited to obtain additional information on the nature of ICT states in Fx, searching for still unidentified spectral features that could contribute to the overall comprehension of the photophysics of this carotenoid and its dark states, a puzzle that still seems to lack some relevant pieces before its completion. 2DES appears to be ideal in this task because of its recognized capability of identifying signatures of dark states through their dynamic coupling with bright states.^42−45^ Moreover, the 10 fs time resolution achieved by this technique is essential to investigate with better detail the ultrafast dynamics subsequent to S_2_ excitation, expected to be in the sub-100 fs time range.^26,28,46,47^

As a carbonyl carotenoid, Fx displays solvent-dependent spectral features. Therefore, we investigated the spectral properties of this carotenoid in solvents with different polarity: methanol (Me), acetone (Ac), and toluene (To). The steady-state absorption spectra of Fx in the three solvents are shown in Figure 1, together with the laser emission profile used for the 2DES experiments. In apolar solvents, like toluene, the typical well-resolved vibronic progression can be identified, while in more polar solvents this structure is progressively lost, and the spectra become broader (spectra in acetone and methanol). It is important to notice that this broadening is asymmetrical, leading to an increase of the intensity on the low-energy red tail of the spectra, especially in methanol. Previous studies on peridinin, another carbonyl carotenoid similar to Fx, suggested that this feature could be related to the charge-transfer character of the ground state, resulting stabilized in polar solvents.^25,26,37^ More recently, Kosumi et al.^31^ proposed that the asymmetric red-shift in methanol is caused by a peculiar conformation of the Fx molecule (“red” form) with strong ICT character.

**Figure 1:**
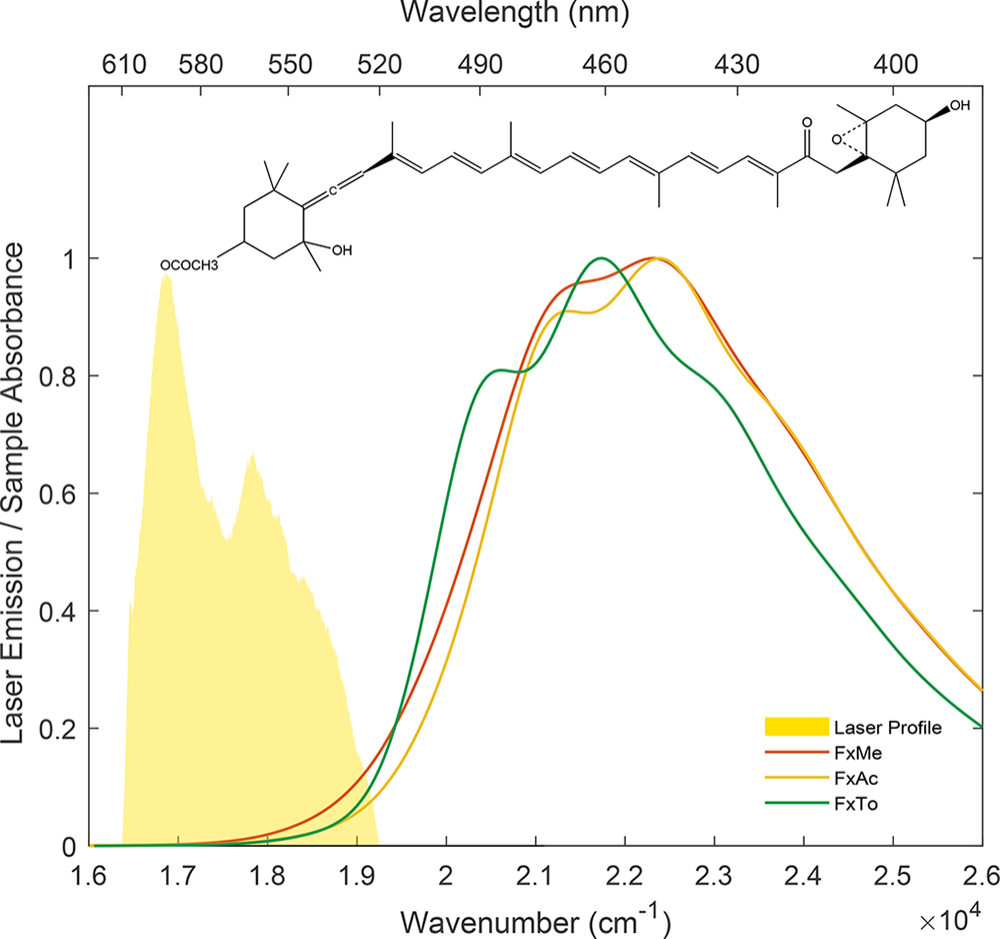
Linear absorption spectra of fucoxanthin in methanol (red line), acetone (yellow line), and toluene (green line), together with the laser emission profile (yellow area). The molecular structure of fucoxanthin is reported in the inset.

The laser profile used in the 2DES measurements covers exactly this spectral region. On the one hand, it is not possible to push the laser spectrum further to blue to cover a bigger portion of the S_0_ → S_2_ transition due to bandwidth limitations of the experimental apparatus used to generate the exciting pulses in the visible range (see the SI). The limitations in the exciting wavelengths prevented so far the systematic characterization of carotenoids by 2DES, as witnessed by the few works available in the literature.^48−52^

On the other hand, however, this configuration focused the investigations only on the red tail of the absorption spectrum, allowing us to better characterize the formation of the ICT state in Fx, the ensuing relaxation dynamics, and its solvent-dependent properties. Moreover, this spectral window also allowed performing a spectral filtering action and neglecting all the relaxation dynamics involving higher energy vibrational levels within the S_2_ manifold. This permitted a significant simplification in the interpretation of the complex dynamics of Fx.

The results of the 2DES experiments, cast into a series of frequency–frequency maps at selected values of population time *t*_2_, are summarized in Figure 2 for the three fucoxanthin samples. The 2DES spectra of Fx are dominated by strong Excited State Absorption (ESA) signals, conventionally reported with a negative sign in 2DES spectroscopy.^44,53^ All the ESA signals are characterized by the same excitation frequency (*x*-coordinate), at around 18900 cm^–1^ (= 529 nm). The *x*-coordinate of the signal reflects what happens to the systems after the first interaction with the laser pulse, i.e., the promotion of the bright S_0_ → S_2_ transition. As previously discussed, this frequency value represents only the red tail of the absorption band, as the laser excitation profile does not cover frequencies higher than 19200 cm^–1^.

**Figure 2:**
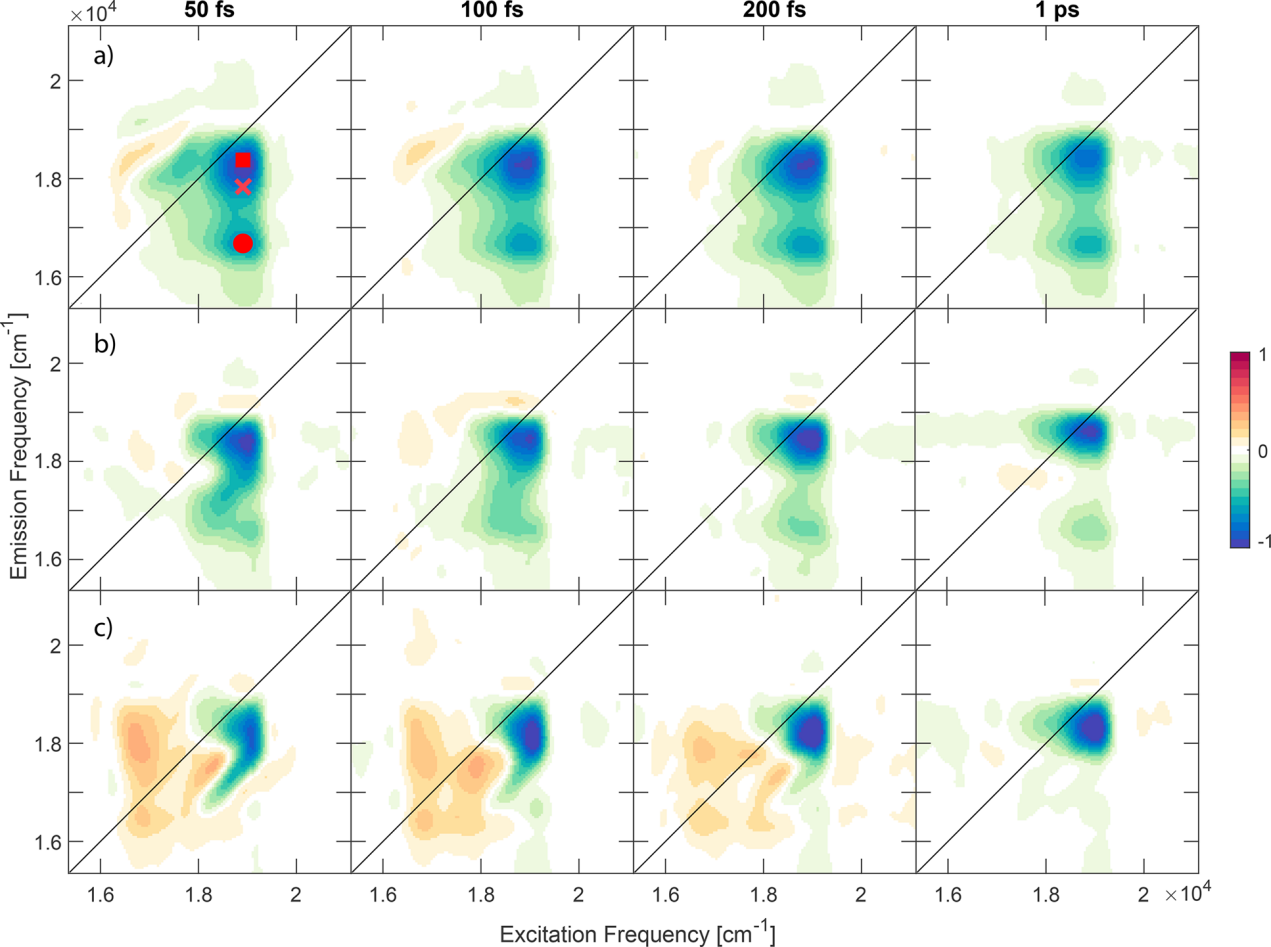
2DES purely absorptive maps of fucoxanthin dissolved in (a) methanol, (b) acetone, and (c) toluene. The square and the circle identify the ESA signals from the S_1_ state and from the ICT state, respectively. The cross indicates the coordinates of the 2D map where the hot vibrational states of S_1_ contribute to the ESA signal.

Fucoxanthin in methanol (FxMe, Figure 2a) displays two distinct ESA signals, one with an emission frequency (*y*-coordinate) of ∼18300 cm^–1^ (square) and the other of ∼16500 cm^–1^ (circle). The presence of two distinct ESA signals in the transient absorption spectra of Fx dissolved in polar solvents has already been captured by pump–probe spectroscopy.^10,28^ The high-energy ESA signal is common to all carotenoids, and it has been attributed to the S_1_ → S*_n_* transition.^26^ The cross in Figure 2a indicates the coordinates where the hot vibrational states of S_1_ are expected to contribute to the ESA signal through the S_2_ → hot S_1_ internal conversion and the hot S_1_ relaxation processes. The band at 16500 cm^–1^, instead, can be found only in Fx and some other carbonyl carotenoids. This band is usually attributed to the presence of an ICT state in the excited states manifold indicating an ESA signal related to the ICT → S_*n*′_ transition.^10,28,29,38^

The response of Fx in acetone (FxAc, Figure 2b) is very similar to the one of FxMe. The main difference is that the lower energy ESA band is substantially less intense. This behavior can be explained through the destabilization of the ICT state in more apolar solvents.^10^ In fact, this solvent-dependent trend is confirmed in the latest set of data collected on Fx in toluene (FxTo, Figure 2c), where the low energy ESA band is not even detected. The positive signals recoded at low excitation and emission frequency coordinates are due to nonresonant solvent contributions.

The evolution of the 2DES maps as a function of the population time has been analyzed through a global complex multiexponential fitting procedure, which allows fitting the dynamic behavior at all the coordinates of the 2D maps simultaneously.^54^ It was demonstrated that this procedure could disentangle in a very efficient way the different components that contribute to the evolution of the 2DES signal and determine with remarkable robustness the kinetic constants regulating the time evolution.^54^ The global fitting methodology was applied to the three sets of data after exclusion of the first 20 fs in order to avoid possible artifacts originating by the time overlap of the exciting pulses.

Figure 3 summarizes the results obtained for the FxAc sample. The results for the other two samples are reported in the SI. The global fitting provides the values of the time constants regulating the relaxation dynamics together with the amplitude distribution of these constants as a function of the excitation and emission frequencies. This amplitude distribution can be visualized in the form of the so-called 2D-DAS (2D-decay associated spectra), associated with each time constant resulting from the fitting (Figure 3a–c).^54^

**Figure 3:**
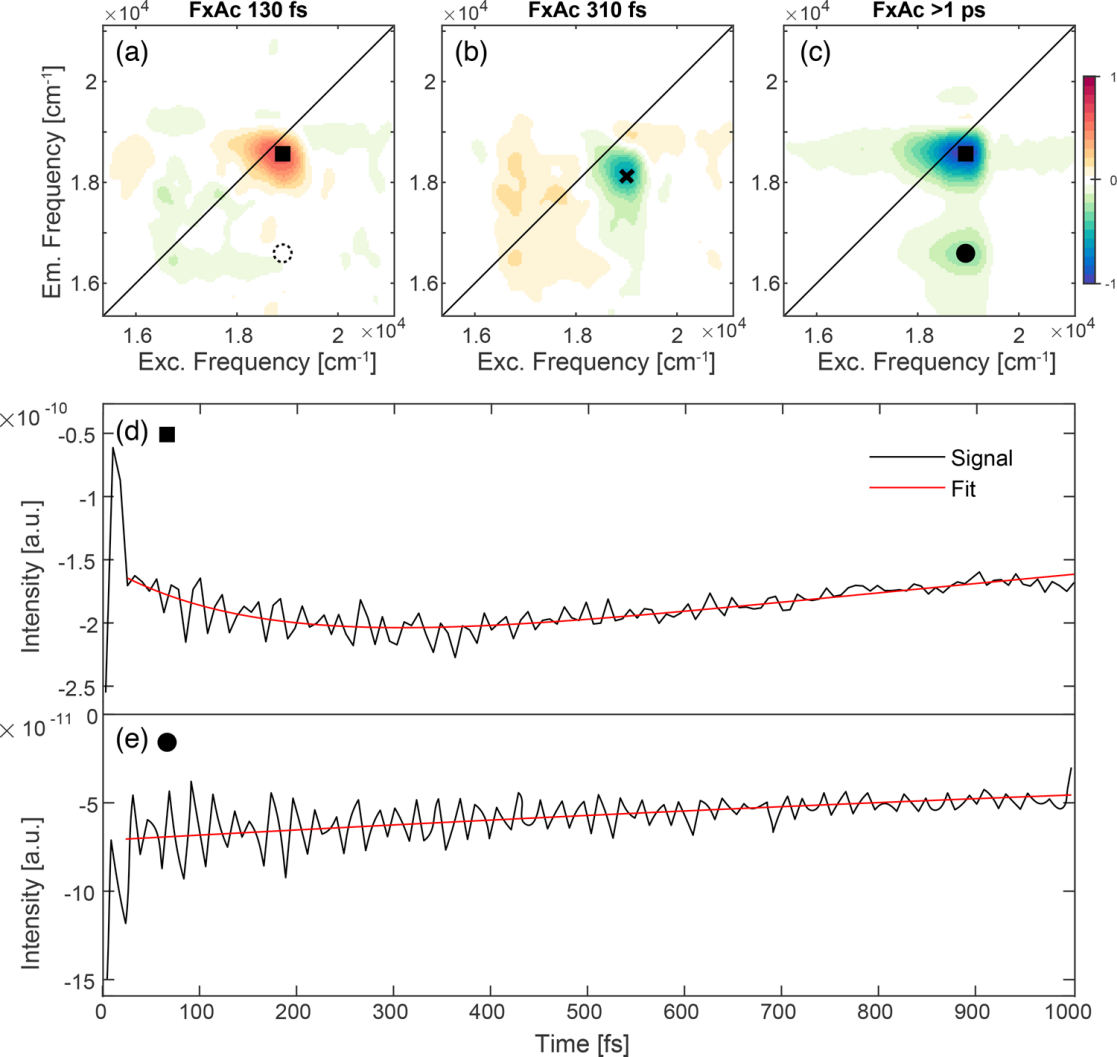
2D-DAS of fucoxanthin in acetone for the three time constants emerged from the fitting and relative traces. The black square (circle) marks the ESA signal from the S_1_ (ICT) state. The cross pinpoints the coordinates where hot S_1_ states are mainly contributing. (a) 2D-DAS of the ultrafast time constant of 130 fs associated with the S_2_ → S_1_ internal conversion. (b) 2D-DAS of the 310 fs component associated with the hot S_1_ relaxation. (c) 2D-DAS of the longer time constant (>1 ps) associated with the S_1_ relaxation. (d) Decay trace showing the *t*_2_ evolution of the signal at the coordinates of the ESA-S_1_ signal (black square). (e) Decay trace at the coordinates of the ESA-ICT signal (black circle).

In the specific case of ESA signals, characterized by a negative amplitude, a positive peak (red) in a 2D-DAS means that, overall, the signal at those coordinates is becoming more negative. This corresponds to a growth of the population of the state from which the ESA originates, and therefore, we refer to this behavior as a “rising” component.^54^ The opposite is true for negative peaks (blue signals in the 2D-DAS), associated with the decay of the population of the same state. These trends can be easily verified by inspecting the time traces at the coordinates of the main peaks appearing in the 2D-DAS, as shown in Figures 3d and 3e.

The 2D-DAS relative to the long-time constant (Figure 3c) captures the main decaying component in both ESA bands, which represents the restoration of the ground state population in the picosecond time scale. The 2D-DAS relative to the shortest time constant (Figure 3a) depicts a rising signal (positive red signal pinpointed by the square), which, considering the sign and the position, can be associated with the S_2_ → S_1_ internal conversion.^29−32^Figure 3b instead is dominated by a decaying component on the lower part of the S_1_-ESA band (blue signal highlighted by the cross). This signal appears at coordinates already associated with hot S_1_ states’ contributions and can been interpreted as the hot S_1_ relaxation, in agreement with other studies.^32^

The same analysis has been applied also to FxMe and FxTo samples. In FxMe, the overall dynamics appeared to be faster with respect to less polar Ac solvent. In this case, it was not possible to identify signatures attributable to the hot S_1_ relaxation, and an overall decay component with a time constant of 65 fs has been found, which we attribute to the S_2_ → S_1_ internal conversion (Figure S4). In the FxTo sample, similar to FxAc, besides the >1 ps long time component, two time constants of 120 and 250 fs have been found. The sign and the amplitude distribution of these time components (Figure S5) suggest a possible attribution to the S_2_ → hot S_1_ and S_2_ → S_1_ processes, respectively. For FxAc (and FxMe), the S_2_ → hot S_1_ process is probably too fast to be clearly characterized.

A closer analysis of the dynamic behavior of the signal at coordinates where hot S_1_ states are expected to contribute (coordinates pinpointed by the cross in Figure 3) highlighted the presence of an additional time component, which could not be reliably distinguished in the global fitting. A local fitting performed at these coordinates (Figure S7) and the comparison with the time decay of FxAc at the same position (Figure S6) lead to the identification of an additional kinetic component with a time constant of 310 fs, which we likely attribute to the hot S_1_ vibrational relaxation. The global fitting could not fully discriminate this component from the 250 fs one, given the small difference between the associated time constants.

The time constants found for the FxTo sample are slightly different than what has been previously found by pump–probe experiments in other nonpolar solvents. For Fx in cyclohexane solutions, Kosumi et al. estimated a time constant of 60 and 620 fs for the S_2_ decay and the S_1_ vibrational relaxation, respectively.^28^ This discrepancy can be explained by accounting for the different nature of the solvent used (toluene rather than cyclohexane) and the different time-resolution (∼10 fs vs ∼100 fs). Nonetheless, the important point is the confirmation of the progressive slowing down of the dynamics in less polar solvents.

Overall, although the relaxation dynamics of S_2_ has been the object of an extensive investigation by pump–probe experiments, the improved time resolution of ∼10 fs achieved with 2DES experiments and the possibility of inspecting the sign and the amplitude distribution of the components in the 2D-DAS plots allowed a better characterization and a robust interpretation of the early steps of relaxation, which also revealed a clear solvent-dependent trend. The obtained time constants for the three samples are summarized in Table 1.

**Table 1:** Time Constants Obtained from the Global Fitting Analysis of the Three Samples^a^

	S_2_ → hot S_1_	S_2_ → S_1_	hot S_1_→ S_1_	S_1_ relaxation
FxMe		65 fs		>1 ps
FxAc		130 fs	310 fs	
FxTo	120	250 fs*	310 fs*	

a The asterisk (*) indicates the time constants that could be discriminated only by a local fitting.

The time trace extracted at coordinates corresponding to the S_1_ → S*_n_* ESA (Figure 3d) shows how the signal evolves in the first 1000 fs after the excitation. The rising and the decay of the ESA band can be observed. Instead, the time trace extracted where the ESA band associated with the ICT → S_*n*′_ transition contributes (Figure 3e) does not present any rising component, and it starts to decay immediately after the laser excitation. This behavior is confirmed by the absence of any rising (red) signals in the corresponding portion of the 2D-DAS (Figure 3a, dashed circle). In addition, both traces reveal the presence of a lively beating behavior, due to the activation of vibrational modes of the Fx. The main beating components in the 2DES signal have been identified through Fourier spectra analysis. Their frequencies agree with well-known characteristic vibrational modes of carotenoids (see the SI).

One of the most important pieces of evidence emerging from the analysis of the three samples is the increase of the rising ultrafast time constant as the polarity of the solvent decreases (Table 1). A similar solvent-dependent trend has been already detected by Kosumi et al.,^30^ but the limited time resolution (ca. 100 fs) did not allow for a detailed discussion of this behavior.

An interesting explanation can be attempted based on a model proposed by Wagner et al.^38^ for peridinin. In this paper, the authors propose the use of an (S_1_+S_2_)/ICT state model, where the ICT state arises from a configurational mixing of the lowest two excited singlet states S_1_ and S_2_. The idea of a mixed state between S_1_ and S_2_ was already proposed in some early works on Peridinin-Chlorophyll Protein (PCP) to explain particular vibrational features.^55,56^ This ICT state is characterized by an enhanced dipole moment and thus requires a polar solvent for stabilization. In this picture, the ICT and the S_1_ states are separated by a polarity-dependent barrier of potential, expected to be very small in polar solvents. The application of a similar model also to Fx would explain the solvent-dependent S_2_ → S_1_ conversion rates: in apolar solvents, the barrier is higher, leading to a longer internal conversion between S_2_ and S_1_.

This model is useful also to discuss the nature of the ICT state itself, focusing on another feature already glimpsed in the analysis of its trace along *t*_2_ (Figure 3d): the absence of a rising time constant related to the ICT-ESA band. In fact, at early delay times, the samples in methanol and acetone share the presence of the ICT-ESA immediately after the laser excitation. The S_1_-ESA band instead is characterized by a rising component, derived from the internal conversion from the S_2_ state. This new experimental evidence could only be captured with a high temporal resolution, and it goes together with recent studies that suggest that S_1_ and ICT are indeed two distinct electronic states, as the temporal evolution of the relative ESA signals is different.^35,36^ The presence of an ESA from the ICT state already at *t*_2_ = 0 suggests an instant population of that state. This evidence has led to the hypothesis that the ICT state could be directly coupled to the S_2_ state, an idea already presented in the (S_1_+S_2_)/ICT state model invoked previously. Indeed, according to Wagner et al.,^38^ “in terms of most properties, the ICT state is S_2_-like in character”. This picture seems to be also supported by the work of Ghosh et al., who identified a <20 fs nonradiative decay of the S_2_ of peridinin to an S_*x*_ state with a strong ICT character, assigned to a distorted configuration.^47,57^

This model refers to peridinin, whose ESA bands are indistinguishable, and it should also be tested for fucoxanthin; but the idea of a strong mixing between the various states of the system could explain this and other controversial features. Moreover, it is important to stress that, even though the S_1_ and the ICT states are two distinct electronic states, an interplay between the two states is still possible, as recent pump-dump-probe studies have proposed.^35,36^ In this picture, more polar solvents, besides stabilizing the ICT and lowering its energy, would also promote a better mixing with S_2_ and thus a lower barrier of potential. Another interesting insight of this model is the interpretation of the asymmetrical increase of the red tail of the linear absorption spectrum in polar solvents: if the ICT state, coupled with the S_2_ state, can be populated instantly, there has to be a trace of this process also in the linear absorption, and this could be indeed related to the increased absorbance in the red tail. In fact, in apolar solvents, the ICT is not populated, and the red tail absorbance is less pronounced. It must finally be noted that all these results have been obtained with very specific excitation conditions, where only the most red portion of the absorption spectrum could be addressed (Figure 1).

In conclusion, in this work, the ultrafast relaxation dynamics of fucoxanthin has been characterized with 2DES, a technique scarcely applied to carotenoid systems because of technical limitations. The obtained results permitted the unraveling of subtle details of the kinetics of the system, through the investigation of the time evolution of ESA signals promoted after the photoexcitation of the red tail of the S_2_ absorption band and the ensuing relaxation to S_1_ and ICT states.

Thanks to the 10 fs time resolution achieved in these experiments, the kinetic constants regulating the S_2_ → S_1_ internal conversion of Fx in three solvents with different polarity have been determined with an unprecedented level of detail. Indeed, it is known that the relaxation dynamics of the S_2_ state in carbonyl carotenoids takes place in the sub-100 fs time regime,^26^ and therefore, conventional pump–probe experiments with a typical 100 fs time resolution so far could only provide rough estimates of the associated kinetic constants. A clear solvent-dependent trend of the S_2_ → S_1_ internal conversion rates has been found: the process becomes faster as the polarity of the solvent increases.

In addition, we found evidence for two distinct ESA signals developing from the S_1_ and ICT states, whose relative intensities also depend on solvent polarity. The S_1_-ESA signal rises in the first 100 fs, which clearly indicates that the S_1_ state is progressively populated via relaxation from S_2_. Instead, the ICT-ESA signal is instantaneously present immediately after photoexcitation, with no rise component, suggesting an instant population of the ICT state. These features have been interpreted on the basis of a model previously proposed in the literature for peridinin, which identified a strong coupling between the ICT and the bright S_2_ state (Figure 4).

**Figure 4:**
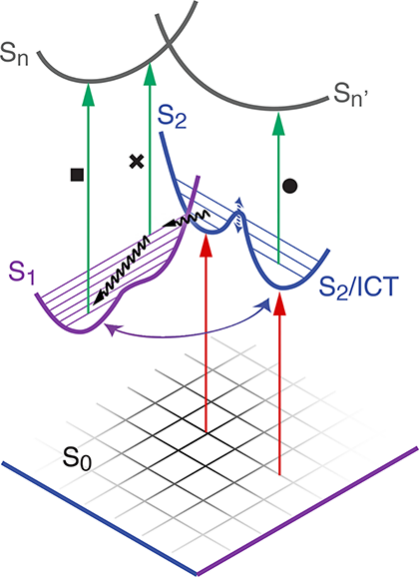
Proposed scheme summarizing the main dynamic processes captured by the 2DES measurements on solutions of Fx. Red arrows: excitation; black wavy arrows: nonradiative processes; green arrows: ESA processes. The black markers (square, cross, and circle) pinpoint the processes identified in the 2D maps of Figures 2 and 3. The barrier of potential between S_2_ and ICT is solvent-dependent (dashed blue arrow). The purple/blue arrow represents the interplay between the S_1_ and ICT states, leading to picosecond equilibration between the potential minima.

While the effective applicability of this model also to fucoxanthin needs to be supported by further studies, the instant presence of the ICT-ESA signal at early times and also its peculiar dependence on solvent polarity point toward a new interpretation of the nature of the ICT state.

These findings represent, in any case, an important piece of information about the nature and the dynamics of dark states in carotenoids. From a broader perspective, we believe that a better characterization of such dynamics is essential for a better comprehension of the ultrafast relaxation dynamics taking place in more complex multichromophoric antenna systems. This preliminary characterization of Fx *in vitro* will also contribute to a deeper understanding of the excitation energy-transfer processes that involve carotenoids *in vivo*.

## Supporting Information Available

Details of 2DES setup and pulse characterization; additional 2DES maps and relative fitting results; and beating analysis (PDF)
