# Supplementary material for: Solvent-Dependent Characterization of Fucoxanthin through 2D Electronic Spectroscopy Reveals New Details on the Intramolecular Charge-Transfer State Dynamics
Source: J Phys Chem Lett. 2021 May 17;12(20):4833–40. doi: 10.1021/acs.jpclett.1c00851 (PMC8279730; doi:10.1021/acs.jpclett.1c00851)
Supplement: Supplementary file 1 — jz1c00851_si_001.pdf [file jz1c00851_si_001.pdf]

SUPPORTING INFO

Solvent-dependent Characterization of Fucoxanthin through 2D Electronic Spectroscopy Reveals New Details on the Intramolecular Charge Transfer State Dynamics

Giampaolo Marcolin,[1] Elisabetta Collini[1*]

[1] *Department of Chemical Sciences, University of Padova, Via Marzolo 1, I-35131 Padova, Italy.*

Contents

S1. 2DES Setup

S2. 2DES Pulse Characterization

S3. Additional 2DES maps

S4. DAS maps for Fucoxanthin in Methanol and Fucoxanthin in Toluene

S5. Additional local fitting results

S6. Beating Analysis

S1. 2DES Setup

2DES measurements have been performed with the setup described in ref [1]. Briefly, a Ti:Sapphire laser system (3 KHz Coherent Libra) generates output pulses at 800 nm, then converted with a non-collinear optical amplifier (NOPA) (Light Conversion TOPAS White) in broad visible pulses. The central wavelength of the broad-band pulses, set at 555 nm, cannot be pushed further towards blue, due to NOPA limitations. A prism compressor system coupled with a pulse shaper (Fastlite Dazzler) permits to get the transform-limited condition for the pulses at the sample position. The setup adopts the fully non-collinear BOXCARS geometry where the laser pulses are split in four identical beams placed on the vertexes of an imaginary square. Three of the resulting beams (the exciting beams) are delayed temporally by pairs of 4° CaF_2 wedges. One wedge of each pair is mounted onto a motorized stage that regulates the thickness of the medium crossed by the beam, modulating the delay times of the pulses with a temporal resolution of 0.07 fs. The fourth beam, instead, is attenuated of 3 orders of magnitude and used as local oscillator (LO) to perform heterodyne detection. The four pulses are then refocused on the sample and, for phase-matching conditions, the signal is emitted on the same direction of the LO pulse. The signal resulting from the interference of the signal and LO is then detected through a spectrograph and an Andor Zyla 5.5 sCMOS camera with 5.5 megapixels.

The pulse duration, optimized through TG-FROG measurements, was compressed to about 10 fs with a spectral bandwidth of about 1470 cm^{-1}. The pulse energy at the sample position was set to 7 nJ per pulse. The outcome of the 2DES experiment is a 3D array of data describing the evolution of 2D frequency-frequency correlation maps as a function of the population time t_2.[2–4] The population time t_2 was scanned from 0 to 1000 fs with steps of 7.5 fs. Each set of measurements was repeated three times to reduce noise and ensure reproducibility. The data have then been analyzed with the global fitting methodology proposed in Volpato *et al.*.[5]

Fucoxanthin was obtained from Sigma-Aldrich® and dissolved in the following anhydrous solvents: methanol, acetone, and toluene. The concentration of the samples was adjusted so to reach an absorbance of ~0.25 in a 1 mm cuvette at 520 nm, leading to a concentration of 0.8 mM. The presence of aggregates in these conditions has been excluded verifying that the absorption spectrum does not significantly vary in a range of concentrations going from 1 μM to 1mM. For each sample, steady-state absorption spectra were acquired before and after each 2DES experiment to verify the absence of degradation of the sample during the measurements.

S2. 2DES Pulse Characterization

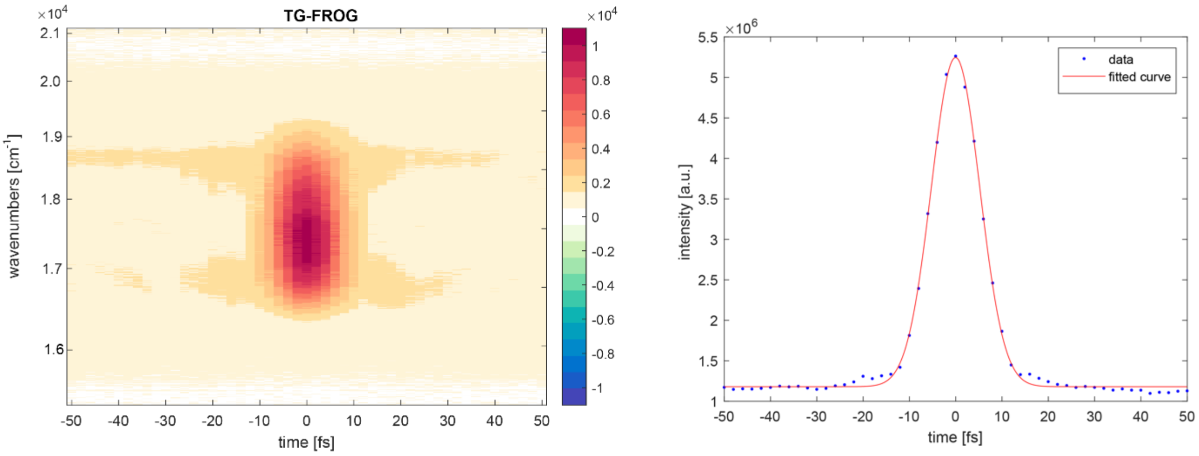

Figure S1. Characterization of the pulse duration through frequency resolved optical gating (FROG) measurements performed on a solvent in the same experimental conditions of the 2DES experiments. (left) FROG plot; (right) Gaussian fit of the FROG signal integrated along the frequency dimension to estimate the pulse duration (8.7 fs).

S3. Additional 2DES maps

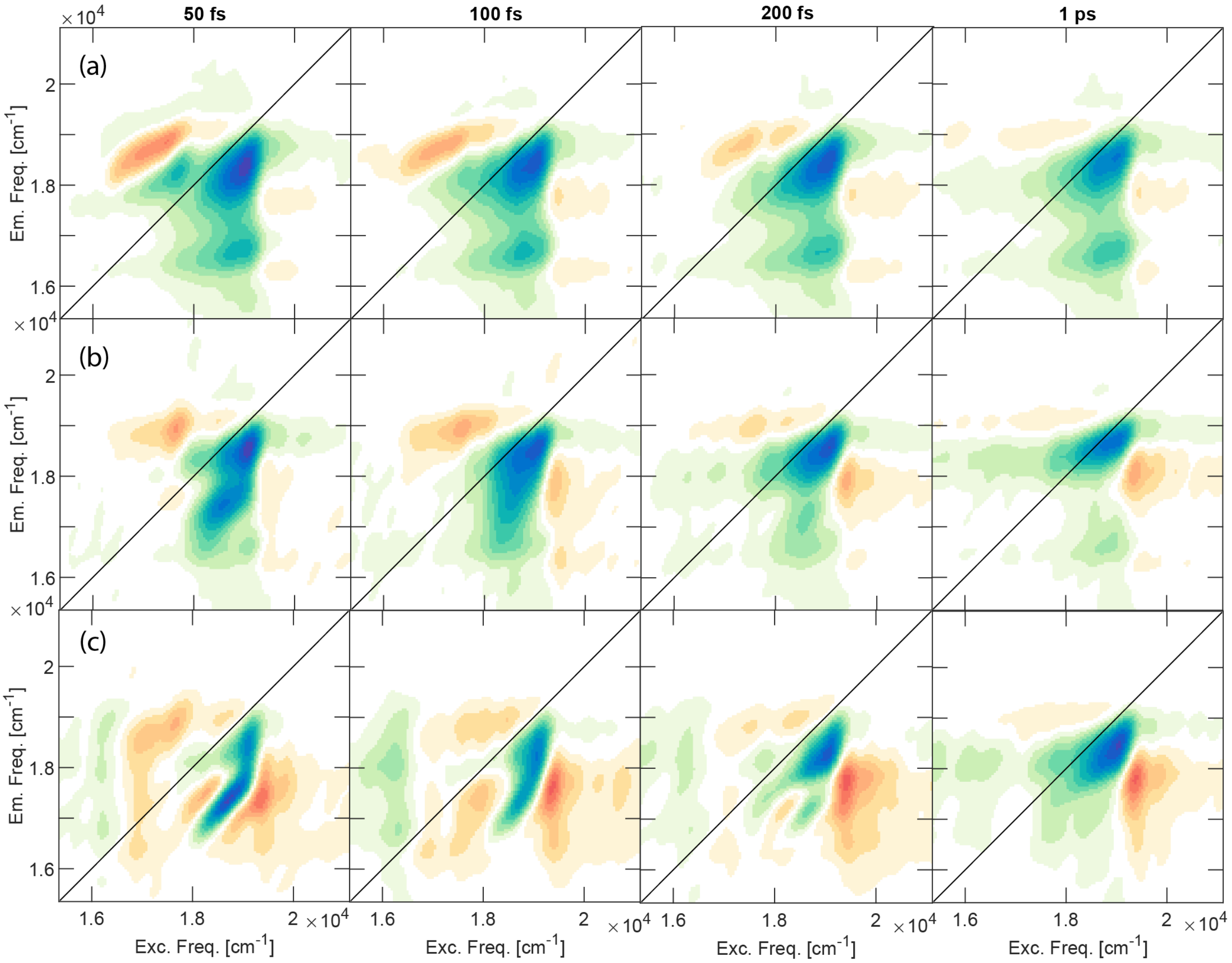

Figure S2. 2DES Rephasing maps of fucoxanthin dissolved in a) methanol; b) acetone; c) toluene.

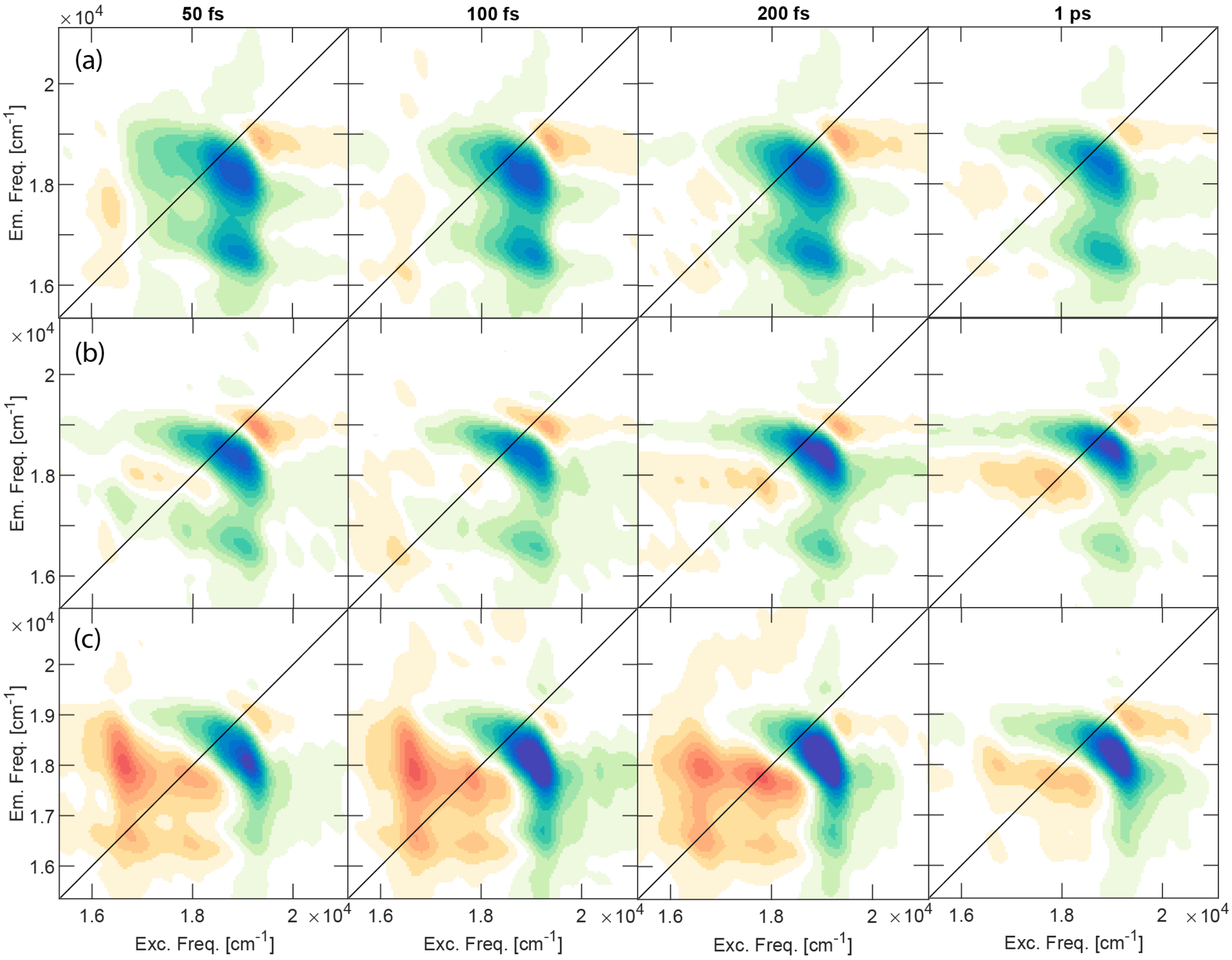

Figure S3. 2DES Non-Rephasing maps of fucoxanthin dissolved in a) methanol; b) acetone; c) toluene.

S4. 2D-DAS maps for Fucoxanthin in Methanol and Toluene

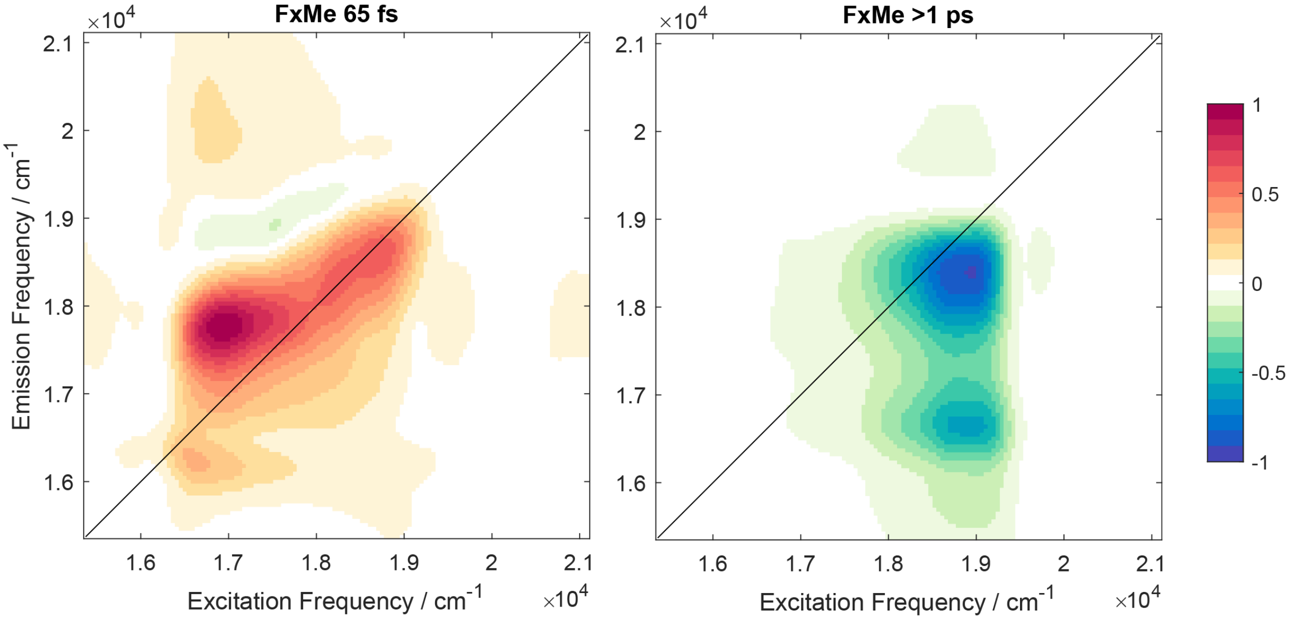

Figure S4. 2D-DAS maps of Fucoxanthin in Methanol

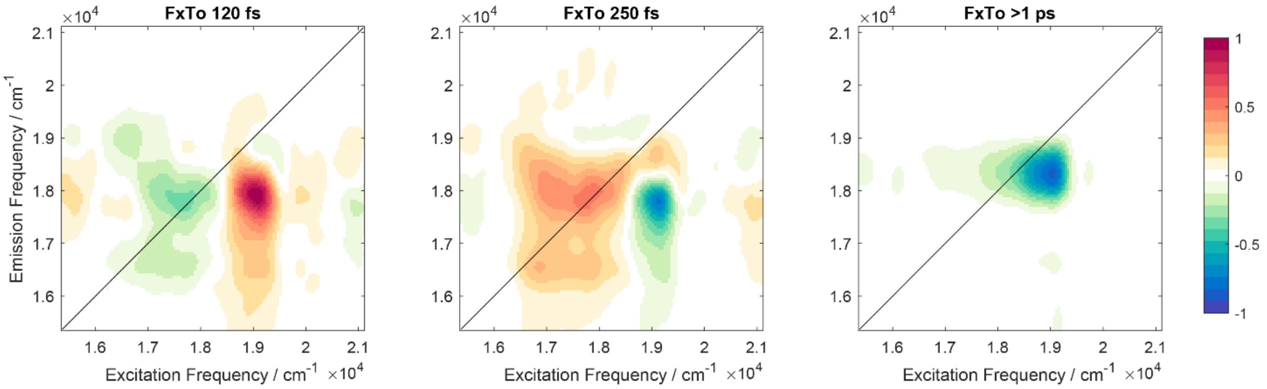

Figure S5. 2D-DAS maps of Fucoxanthin in Toluene

S5. Additional local fittings results

Local fittings based on a 3-exponential model have been performed at specific coordinates to untangle the multiple dynamics taking place in hundreds fs time scale.

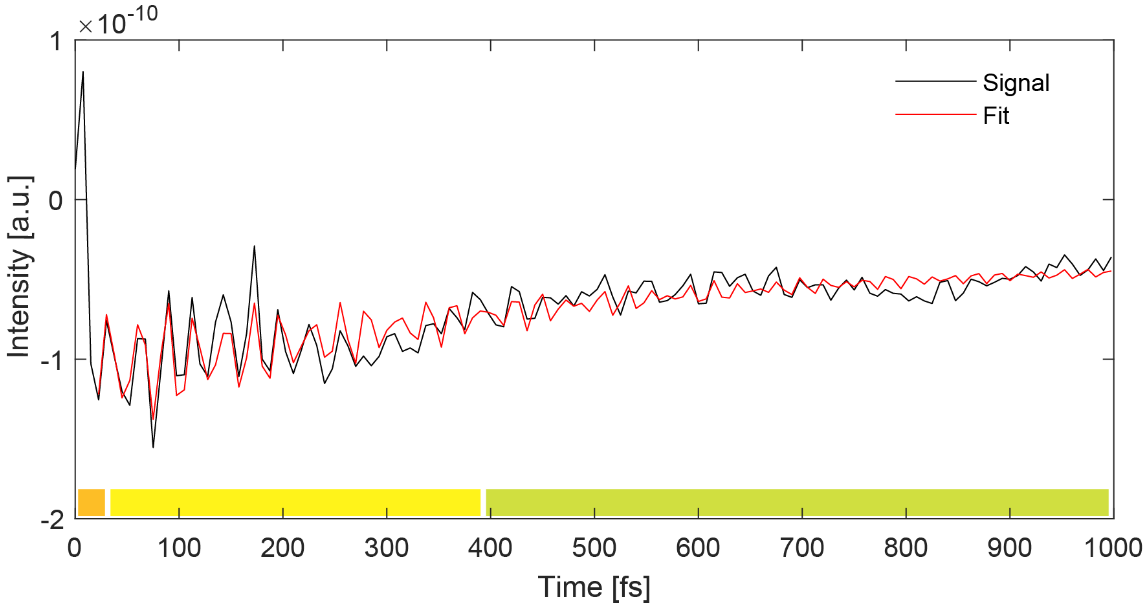

Figure S6. Decay trace showing the t_2 evolution of the signal at the coordinates 19000, 17800 cm^{-1} for the FxAc sample. The three colored bars indicate the time ranges for the three processes that govern this evolution: the rising of the signal associated to the $S_2 \rightarrow$ hot S_1 internal conversion (orange), the fast decay associated to the hot S_1 relaxation (yellow), and the slow decay as the population is restored to the ground state (green). The corresponding time constants are: < 10 fs, 310 fs and > 1 ps, respectively

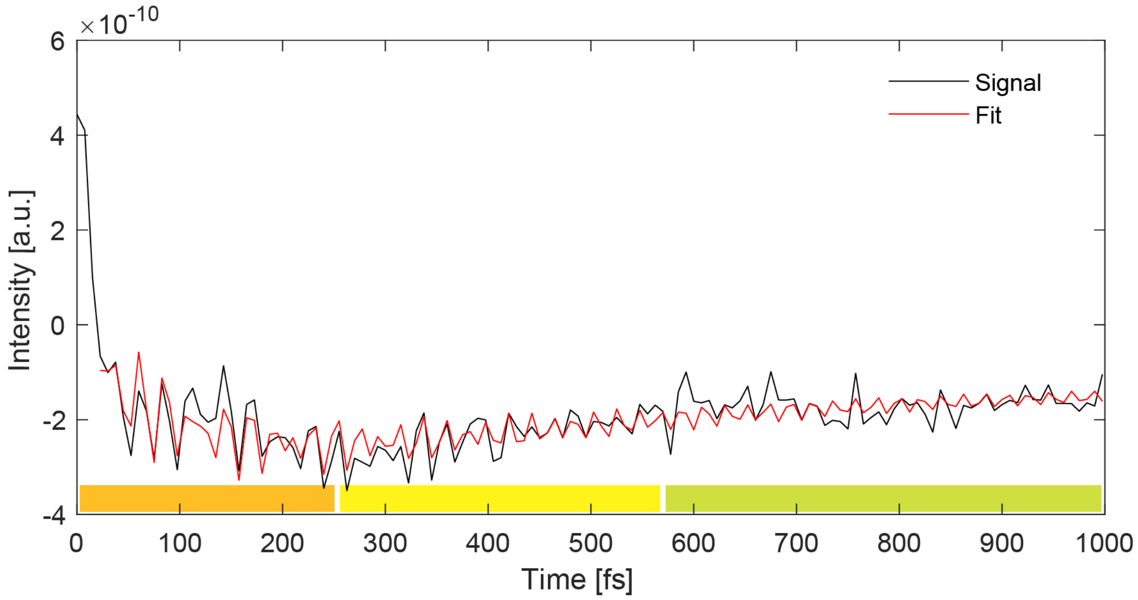

Figure S7. Same as Figure S6 for FuTo sample. The corresponding time constants are: 120 fs, 310 fs and > 1 ps.

S6. Beating Analysis

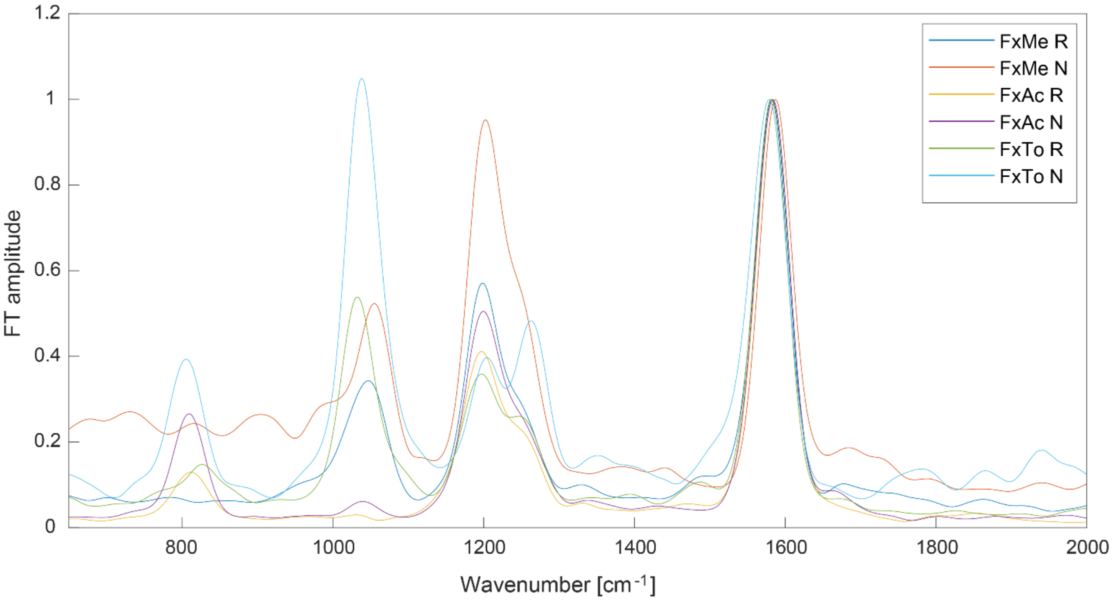

Figure S8. Fourier spectrum analysis of the beatings in the t_2 evolution of the signal for the three samples (R=rephasing, N=non rephasing). The main beating components correspond to vibrational modes of Fx (~1200, ~1280, ~1580 cm^{-1}).[6,7] Signals below 1200 cm^{-1} are due to non-resonant solvent contributions: the main peak of methanol is centered at ~1050 cm^{-1} and the one of acetone at ~800 cm^{-1}. Toluene, instead, has two main components at ~800 cm^{-1} and ~1030 cm^{-1}.

References

(1) Bolzonello, L.; Volpato, A.; Meneghin, E.; Collini, E. Versatile Setup for High-Quality Rephasing, Non-Rephasing, and Double Quantum 2D Electronic Spectroscopy. *J. Opt. Soc. Am. B* **2017**, *34* (6), 1223–1233. https://doi.org/10.1364/josab.34.001223.

(2) Bránczyk, A. M.; Turner, D. B.; Scholes, G. D. Crossing Disciplines - A View on Two-Dimensional Optical Spectroscopy. *Ann. Phys. (Berlin)* **2014**, *526* (1–2), 31–49. https://doi.org/10.1002/andp.201300153.

(3) Collini, E. Spectroscopic Signatures of Quantum-Coherent Energy Transfer. *Chem. Soc. Rev.* **2013**, *42* (12), 4932–4947. https://doi.org/10.1039/c3cs35444j.

(4) Gelzinis, A.; Augulis, R.; Butkus, V.; Robert, B.; Valkunas, L. Two-Dimensional Spectroscopy for Non-Specialists. *Biochim. Biophys. Acta* **2019**, *1860* (4), 271–285. https://doi.org/10.1016/j.bbabio.2018.12.006.

(5) Volpato, A.; Bolzonello, L.; Meneghin, E.; Collini, E. Global Analysis of Coherence and Population Dynamics in 2D Electronic Spectroscopy. *Opt. Express* **2016**, *24* (21), 24773. https://doi.org/10.1364/oe.24.024773.

(6) Rimai, L.; Heyde, M. E.; Gill, D. Vibrational Spectra of Some Carotenoids and Related Linear Polyenes. A Raman Spectroscopic Study. *J. Am. Chem. Soc.* **1973**, *95* (14), 4493–4501.

(7) Merlin, J. C. Resonance Raman Spectroscopy of Carotenoids and Carotenoid-Containing Systems. *Pure Appl. Chem.* **1985**, *57* (5), 785–792.
